# Supplementary material for: Exploring the gender gap in young adult mental health during COVID-19: Evidence from the UK
Source: PLoS One. 2024 Dec 19;19(12):e0305680. doi: 10.1371/journal.pone.0305680 (PMC11658509; doi:10.1371/journal.pone.0305680)
Supplement: S1 Appendix — (DOCX) [file pone.0305680.s001.docx]

**S1: Appendix A: Source data for each Survey wave**

| **Wave** | **Source Data** | **Month/Year** |
| --- | --- | --- |
| 1 | UKHLS mainstage survey- wave 10/wave 11 | Collected from January 2019-May 2021. Only data collected in 2019 is included as a baseline. |
| 2 | UKHLS COVID-19 Survey- wave 1 web survey | April 2020 |
| 3 | UKHLS COVID-19 Survey- wave 2 web survey | May 2020 |
| 4 | UKHLS COVID-19 Survey- wave 3 web survey | June 2020 |
| 5 | UKHLS COVID-19 Survey- wave 4 web survey | July 2020 |
| 6 | UKHLS COVID-19 Survey- wave 5 web survey | September 2020 |
| 7 | UKHLS COVID-19 Survey- wave 6 web survey | November 2020 |
| 8 | UKHLS COVID-19 Survey- wave 7 web survey | January 2021 |
| 9 | UKHLS COVID-19 Survey- wave 8 web survey | March 2021 |
| 10 | UKHLS COVID-19 Survey- wave 9 web survey | September 2021 |
