# Supplementary material for: Exploring the gender gap in young adult mental health during COVID-19: Evidence from the UK
Source: PLoS One. 2024 Dec 19;19(12):e0305680. doi: 10.1371/journal.pone.0305680 (PMC11658509; doi:10.1371/journal.pone.0305680)
Supplement: S2 Appendix — (DOCX) [file pone.0305680.s002.docx]

**S2 Appendix B: GHQ-12 Survey Question Design**

Questions are presented to the participants in bold, with a selection of responses in brackets below.

“The next questions are about how you have been feeling recently…

1. **Have you recently been able to concentrate on whatever you're doing?**

{Better than usual, Same as usual, Less than usual, Much less than usual}

1. **Have you recently lost much sleep over worry?**

{Not at all, No more than usual, Rather more than usual, Much more than usual}

1. **Have you recently felt that you were playing a useful part in things?**

{More so than usual, Same as usual, Less so than usual, Much less than usual}

1. **Have you recently felt capable of making decisions?**

{More so than usual, Same as usual, Less so than usual, Much less capable}

1. **Have you recently felt constantly under strain?**

{Not at all, No more than usual, Rather more than usual, Much more than usual}

1. **Have you felt you couldn’t overcome your difficulties?**

{Not at all, No more than usual, Rather more than usual, Much more than usual}

1. **Have you recently been able to enjoy your normal day-to-day activities?**

{More so than usual, Same as usual, Less so than usual, Much less than usual}

1. **Have you recently been able to face up to problems?**

{More so than usual, Same as usual, Less so than usual, Much less able}

1. **Have you recently been feeling unhappy or depressed?**

{Not at all, No more than usual, Rather more than usual, Much more than usual}

1. **Have you recently been losing confidence in yourself?**

{Not at all, No more than usual, Rather more than usual, Much more than usual}

1. **Have you recently been thinking of yourself as a worthless person?**

{Not at all, No more than usual, Rather more than usual, Much more than usual}

1. **Have you recently been feeling happy, all things considered?**

{More so than usual, About the same as usual, Less so than usual, Much less than usual}
