# Supplementary material for: Exploring the gender gap in young adult mental health during COVID-19: Evidence from the UK
Source: PLoS One. 2024 Dec 19;19(12):e0305680. doi: 10.1371/journal.pone.0305680 (PMC11658509; doi:10.1371/journal.pone.0305680)
Supplement: S3 Appendix — (DOCX) [file pone.0305680.s003.docx]

**S3 Appendix C: Missing Data**

| **Demographic** | **Sample missing n (%)** |
| --- | --- |
| Sex | 91 (0.06%) |
| Age | 3 (0.00%) |
| Ethnicity | 845 (0.55%) |
| Higher Education | 1,154 (1.01%) |
| Relationship Status | 257 (0.17) |
| Employment Status | 7 (0.00) |
| Household Composition | 0 (0%) |
| Region | 42 (0.03%) |
| Location (Urban/ Rural) | 1,877 (1.21%) |
| Disability | 333 (0.22) |
| Loneliness | 3,078 (1.99%) |
| Hours spent on childcare | 1 (0.00%) |
| Hours spent on cleaning | 10,646 (6.89%) |
| GHQ | 7,028 (4.55%) |

Note: Missing data includes actual missing data (i.e. non-completion of the survey), refusal and don’t know responses.
