# Supplementary material for: Exploring the gender gap in young adult mental health during COVID-19: Evidence from the UK
Source: PLoS One. 2024 Dec 19;19(12):e0305680. doi: 10.1371/journal.pone.0305680 (PMC11658509; doi:10.1371/journal.pone.0305680)
Supplement: S4 Appendix — (DOCX) [file pone.0305680.s004.docx]

**S4 Appendix D: Random-effects regression subgroup analysis of the impact of loneliness and domestic time use on GHQ**

|  | **Young adults (<25)** | **Older adults (25+)** | **Young adults (<25)** | **Older adults (25+)** |
| --- | --- | --- | --- | --- |
|  | β (sd.error) | β (sd.error) |  |  |
| **Loneliness**  Sometimes  Often  **Care hours**  1-15  15 +  **Clean hours**  6-10  10+  **Constant** | 3.67 (0.13)***  9.38 (0.18) ***  10.35 (0.11) *** | 3.24 (0.03) ***  8.29 (0.06)***  10.59(0.29) *** | 0.2 (0.26)  0.74 (0.32) *  -0.01 (0.18)  0.29 (0.25)  13.79 (0.19) *** | 0.22 (0.06) ***  0.39 (0.07) ***  -0.08 (0.04) *  0.06 (0.04)  12.09 (0.06) *** |
| **Observations**  **R2** | 8,686  0.33 | 126,620  0.32 | 6,768  0.001 | 111,076  0.009 |
