# Supplementary material for: Exploring the gender gap in young adult mental health during COVID-19: Evidence from the UK
Source: PLoS One. 2024 Dec 19;19(12):e0305680. doi: 10.1371/journal.pone.0305680 (PMC11658509; doi:10.1371/journal.pone.0305680)
Supplement: S5 Appendix — (DOCX) [file pone.0305680.s005.docx]

**S5 Appendix E: Framework for regression model**

GHQ_ij_ = β_0_ + β_1_ (Female) _ij_ + β_2_ (Young) _ij_ + β_3_ (Young Female) _ij_ + β_4_ (Loneliness) _ij_  + β_5_ (Carehrs) _ij_ + β_6_ (Cleanhrs) _ij_ + β_7_ (Ethnicity) _ij_ + β_8_(education) _ij_ + β_49_(relationship status) _ij_ + β_10_ (employment status) _ij_ + β_11_ (UK region) _ij_  + β_12_ (urban) _ij_ + β_13_ (household composition) _ij_   + β_14_ (disability) _ij_  + β_15_(wave) _ij_  + (µ_ij_ + ε_i_)

Where GHQ is the outcome variable, _i_ is time and _j_ is the individual. β_0_ is the constant and µ_ij_ + ε_i_ correspond to error terms for both the individual and time clustering. Female is a binary for being female, young is a binary variable for being 16-24, and Young Female is a binary variable for being a woman between the ages of 16-24. Ethnicity is a binary variable for being non-white, urban is a binary variable for living in an urban area and disability is a binary variable for having a long-term limiting illness. Reference categories: Hardly or Never lonely, 0 hours of childcare per week, 0-6 hours of cleaning per week, having a degree, being in employment, being single, living in England, living alone and wave 8 of data collection (Jan 2021).
