# Supplementary material for: Exploring the gender gap in young adult mental health during COVID-19: Evidence from the UK
Source: PLoS One. 2024 Dec 19;19(12):e0305680. doi: 10.1371/journal.pone.0305680 (PMC11658509; doi:10.1371/journal.pone.0305680)

**S7 Appendix G: Proportion of loneliness, hours spent on childcare and hours spent on cleaning by age and gender**


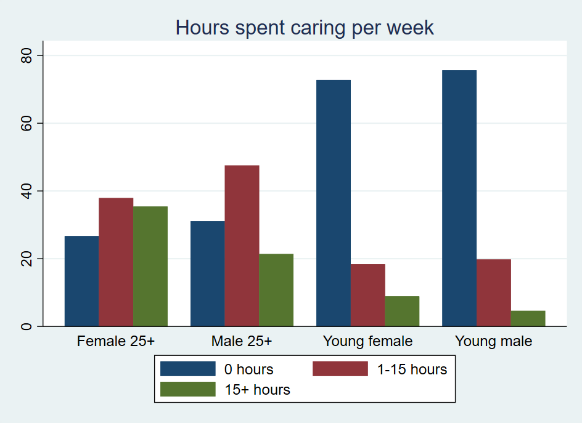

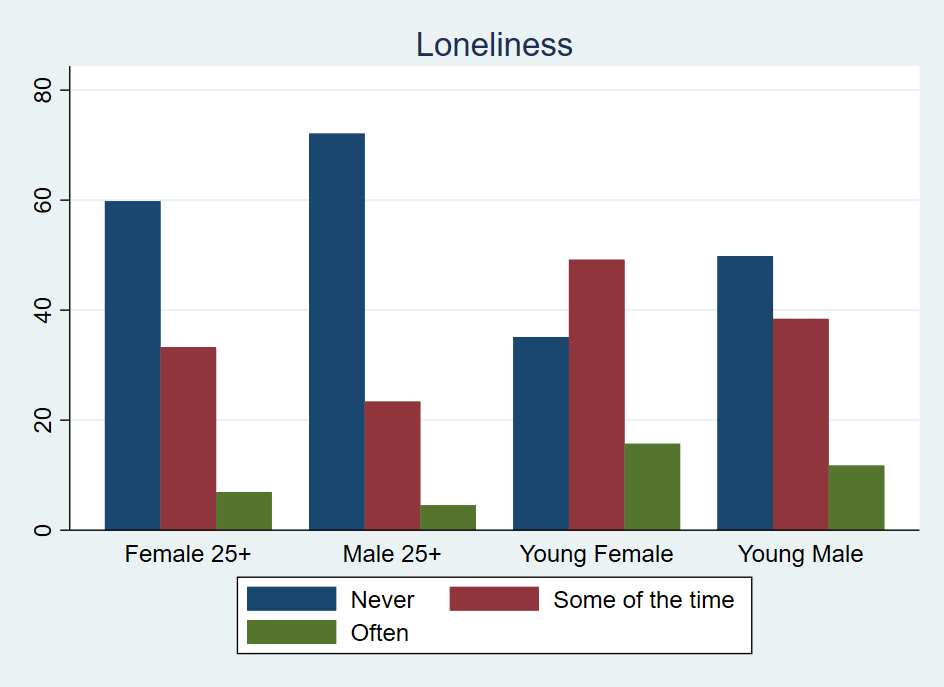

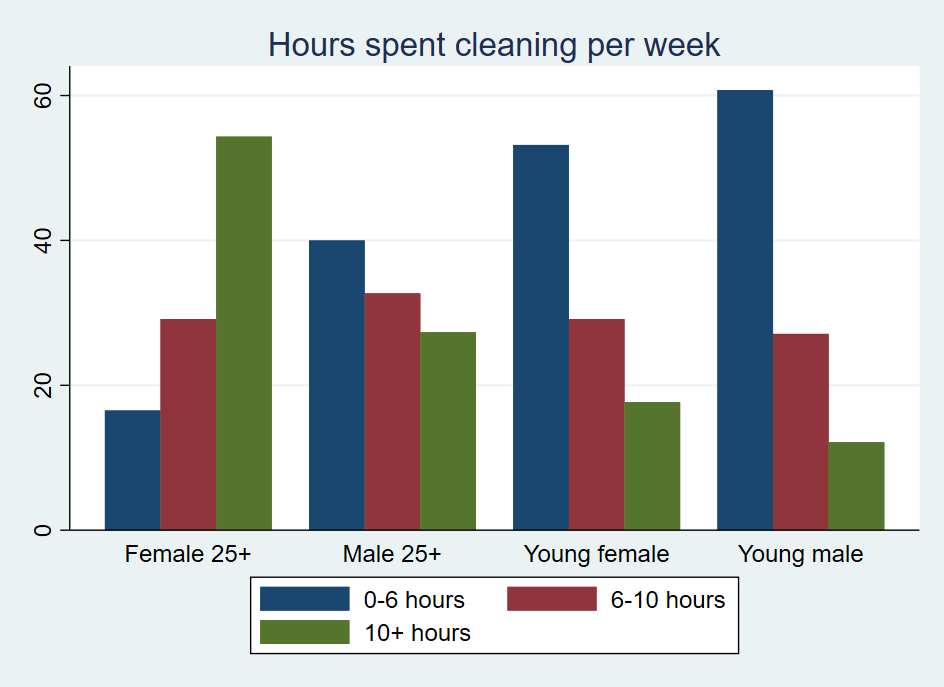

Supplement: S7 Appendix — (DOCX) [file pone.0305680.s007.docx]
